# Supplementary material for: Construction of an automated machine learning-based predictive model for postoperative pulmonary complications risk in non-small cell lung cancer patients undergoing thoracoscopic surgery
Source: PLoS One. 2025 Sep 26;20(9):e0333413. doi: 10.1371/journal.pone.0333413 (PMC12469157; doi:10.1371/journal.pone.0333413)
Supplement: Attachment — Submitted filename: Response to Reviewers.docx [file pone.0333413.s001.docx]

**Response to Reviewers**

Reviewer #1: It was a great pleasure to review the manuscript titled “Construction of an Automated Machine Learning-Based Predictive Model for Postoperative Pulmonary Complications Risk in Non-small Cell Lung Cancer Patients Undergoing Thoracoscopic Surgery” by Xie Qiu et al. In this study, the authors proposed a predictive model for postoperative pulmonary complications (PPCs) using automated machine learning (AutoML), and they further developed a platform to facilitate clinical application of the model. The study addresses an important clinical need, and the methodology is clearly described with appropriate figures and statistical analyses. However, there are several points that require clarification or improvement before the manuscript can be considered for publication.

Concerns:

#1. Regarding the occurrence of postoperative pulmonary complications (line 105-106, line 138-140): The authors included pneumonia, pleural effusion, atelectasis, pneumothorax, and persistent air leak as components of postoperative pulmonary complications (PPC). However, the severity of these complications is not clearly defined. For instance, it is unclear how long the air leak persisted or whether invasive therapeutic interventions were required for its management. The Clavien-Dindo classification is widely accepted for grading the severity of postoperative complications, with Grade 3 or higher indicating the need for invasive procedures, such as reoperation, CVC placement, and drainage tube insertion. Clarifying the severity of PPCs using standardized classification system would enhance the clinical relevance of the findings, particularly since mild complications (e.g., Clavien-Dindo Grade 1 or less) may not have significant clinical implications.

Dear Reviewer,

Thank you for your insightful comments and constructive feedback regarding the definition of postoperative pulmonary complications (PPC) in our manuscript (Lines 105–106, 138–140). We acknowledge the validity of your point on the lack of severity grading for included PPCs (pneumonia, pleural effusion, atelectasis, pneumothorax, and persistent air leak), as illustrated by ambiguities such as the duration of air leaks or requirement for invasive management. We concur that standardized systems like the Clavien-Dindo classification—with Grade 3+ indicating invasive interventions such as reoperation or drainage—would enhance clinical relevance by distinguishing benign events (e.g., Grade 1) from those with significant implications.

In this study, our primary objective was to develop a predictive model using a binary outcome of "any PPC vs. no PPC," as defined by the occurrence of one or more complications detailed in our cohort analysis. This approach aligns with our focus on identifying patients at risk for any PPCs to enable early clinical decision-making, a scope reflected in our feature engineering and algorithm design. However, we recognize that incorporating severity stratification (e.g., Clavien-Dindo) would provide deeper clinical insights, particularly for differentiating high-impact complications.

To address your comment:

We have revised the Methods section to explicitly state: "PPCs were assessed as a binary endpoint (present or absent) based on predefined criteria, without severity grading," citing limitations in our original design.

In the Limitations, we added a description of this limitation as well as the research we are doing.

Your critique greatly strengthens the manuscript's scientific rigor. We appreciate your valuable expertise!

#2. Regarding the Baseline Characteristics of training and testing cohorts (line 143): The authors provided a comprehensive set of baseline characteristics; however, operative time was not included in the analysis. Recent studies have demonstrated that the prolonged operative time is associated with postoperative complications following lung surgery (Paolo de Angelis et al., Ann Surg. 2023 December 01, 278(6): e1259-e1266). Given this evidence, the inclusion of operative time as a variable in the predictive model may enhance its accuracy and clinical relevance.

Dear Reviewer,

Thank you for your valuable comments regarding the inclusion of operative time as a predictor variable (line 143). We appreciate your reference to de Angelis et al.’s seminal work (Ann Surg. 2023) on operative duration and pulmonary complications.

In response:

Supplementary Data: We have added operative time to the baseline characteristics in Table 1 (see revised Results, Section 3.1).

Explanation for Exclusion: As detailed in the revised Discussion, operative time was considered during model development but failed to demonstrate predictive significance. This may partly result from: (1) Sample Constraints: A total cohort of 286 patients may lack power to capture nuanced relationships; (2) Imputation Limitations: Mean substitution for missing values could mask clinical thresholds (e.g., complications specific to >4-hour procedures).

Commitment: We concur this variable merits deeper investigation. Our upcoming work will integrate exact operative metrics via wearable sensors to overcome current data gaps.

These revisions strengthen our Discussion section while honoring your clinical expertise.

#3. In general, the topic is relevant to clinicians, who are not familiar with highly specialized methods such as BLSO and the enhancement methods used. A brief explanation may assist these readers.

Dear Reviewer,

Thank you for your insightful comment regarding the need for enhanced methodological accessibility for clinicians. We fully acknowledge that specialized algorithms like IBSLO may present comprehension barriers. In direct response:

(1) Structural Revisions: Section 2.3 was comprehensively restructured to clarify the IBSLO-driven AutoML framework. We now emphasize: Clinically tangible analogies ("biologically inspired search strategy") for IBSLO's optimization mechanics; Explicit prioritization of clinical outcomes over technical minutiae, particularly highlighting: "The IBSLO-guided AutoML prioritizes clinically actionable outputs—identifying critical predictors like preoperative leukocyte count and BMI—without demanding algorithmic expertise";
(2) Concise Benchmarking: Technical validation (CEC2022 functions) was condensed to a single sentence with visual support: "IBSLO demonstrated superior convergence and stability versus conventional algorithms, ensuring reliable clinical application";
(3) Clinical Translation: A new concluding paragraph explicitly states: "Clinicians can utilize our APP for PPC risk stratification without engaging with underlying algorithmic complexity"

These adjustments ensure methodological transparency while maintaining clinician-centric focus, aligning with your recommendation.

#4. Regarding study design (line 109-110): What is the final model that autoML has selected, how were the features selected, what kind of hyper-parameters were used? If this is not relevant to your methodology using ISBLO, please state the reason.

Dear Reviewer,

Thank you for your constructive feedback regarding the AutoML methodology in our study design (line 109-110). As detailed in Sections 2.3 and 3.3 of our revised manuscript, the AutoML framework driven by the Improved Blood-Sucking Leech Optimizer (IBSLO) selected an optimized XGBoost model as the final predictive architecture, based on its superior performance in capturing nonlinear interactions specific to postoperative complications. Feature selection was intrinsically governed by IBSLO’s binary-encoded solution vector, which automatically identified and prioritized six clinically significant predictors: preoperative leukocyte count, BMI, surgical approach, age, intraoperative blood loss, and C-reactive protein levels, with SHAP analysis further validating their clinical relevance and weighting. Hyperparameter optimization was unified within IBSLO’s metaheuristic search space, dynamically tuning key parameters such as XGBoost’s learning rate, max_depth, and gamma through opposition-based learning strategies to avoid local optima while maintaining computational efficiency. Crucially, IBSLO’s role was fundamental to this process—it seamlessly integrated model selection, feature subspace identification, and hyperparameter tuning into a single optimization paradigm, overcoming fragmentation issues inherent in traditional AutoML workflows, with its enhanced convergence stability having been rigorously validated against five competing algorithms across 12 CEC2022 benchmark functions. We have explicitly clarified this IBSLO-AutoML synergy in Section 2.3 and invite your attention to Table 2 (Section 3.3) for quantifiable architectural details.

#5. Can you explain how autoML with IBSLO contributed to ‘Robustness against data heterogeneity’? Did it also contribute to minimum overfitting despite the small sample size?

Dear Reviewer,

Thank you for raising this important methodological consideration. The IBSLO-driven AutoML framework achieves robustness against data heterogeneity through its hierarchical learner integration architecture, which dynamically orchestrates linear models for structural coherence and noise tolerance alongside gradient-boosted trees that capture localized nonlinear patterns—enabling adaptive responses to imbalanced distributions and variable interaction complexities across our demographic, metabolic-inflammatory, and surgical data domains. This versatility directly addresses the pitfalls of monolithic algorithms when processing high-dimensional clinical datasets with embedded heterogeneity, as noted in prior literature on traditional optimization approaches converging to local optima. Regarding overfitting mitigation in small samples, IBSLO employs dual protective mechanisms: Explicit feature sparsity regularization during fitness evaluation penalizes excessive model complexity, while embedded dimensionality reduction systematically eliminates redundant predictors via population-based search operators—a strategy validated by the model's consistent performance (training AUC=0.9038 vs. test AUC=0.8775) despite our cohort size. These defenses are further reinforced by our strict isolation protocols: nested ten-fold cross-validation was exclusively performed on training partitions, and solution vectors were evolved without exposure to test sets. Quantitative validation through 30 independent IBSLO runs demonstrated minimal performance degradation (average AUC variance <0.018) even when introduced to hidden heterogeneity in surgical patterns or inflammatory biomarker distributions.

We have added a discussion description in the discussion section.

#6. Please clarify that feature selection and cross-validation were done after train-test splitting.

Dear Reviewer,

Thank you for highlighting this crucial methodological detail. We confirm that strict temporal separation was maintained: the dataset was first stratified into independent training (80%, n=229) and testing sets (20%, n=57) prior to any downstream procedures. Feature selection via IBSLO and subsequent hyperparameter optimization were conducted exclusively within the training subset using ten-fold cross-validation, preventing any information leakage to the test set. This ensured that all feature ranking procedures (including SHAP-derived importance weights) and final model validation were derived solely from the training cohort. Cross-validation served as an embedded regularization mechanism during the metaheuristic search phase, iteratively refining feature subspaces without accessing test data. Only after final model freezing were truly blinded predictions generated for the test set. We have clarified this sequence explicitly in Section 2.3.

Minor Points:

#1. The legend of blue bar should be changed from BSLO to IBSLO in Figure 2.

Dear Reviewer,

Thank you for identifying this critical labeling inconsistency. We confirm that the legend entries mislabeled as "BSLO" in Figure 2 resulted from an erroneous configuration during initial visualization scripting, where the improved algorithm's (IBSLO) designation was accidentally truncated to the base algorithm's name (BSLO) in two subplots. This naming inconsistency occurred at the plotting interface-level and did not affect the underlying computational protocols or IBSLO's validated performance advantage across CEC2022 functions (Figure 2 remains an accurate illustration of IBSLO's accelerated convergence and reduced susceptibility to local optima versus BSLO/other algorithms). To remedy this: 1) All twelve benchmark function visualizations were re-audited to replace legacy "BSLO" labels with "IBSLO" annotations; 2) Scripts were reconfigured with explicit naming protocols distinguishing IBSLO from its predecessor; 3) Corrected high-resolution figures were regenerated from source data using validated matrix parameters. These adjustments ensure precise correspondence between experimental design and graphical representation. We deeply regret any confusion caused by this presentational oversight.

Reviewer #2: Sun et al. implemented an integrative predictive framework that utilizes machine learning and clinical parameters to predict postoperative pulmonary complications in patients with non-small cell lung cancer. Overall, the manuscript addresses a significant research gap in assessing the predictive model for postoperative complications among patients with NSCLC by leveraging AutoML. However, there are several areas where the manuscript can be improved. I have suggested improvements to enhance the clarity of the reporting results and format.

1.In abstract, the abbreviation of IBSLO should be revised.

Dear Reviewer,

Thank you for your meticulous attention to terminological precision. We confirm that in the revised abstract, the first mention of the algorithm now appears as "Improved Blood-Sucking Leech Optimizer (IBSLO)" when introduced in the methods statement, with subsequent references using the abbreviated "IBSLO" consistently. This modification ensures terminological integrity by explicitly establishing both the expanded nomenclature and standardized abbreviation upon initial occurrence, aligning with journal conventions. The revised phrasing precisely reflects our methodological distinction between the base BSLO architecture and its enhanced variant featuring Cubic mapping initialization and dynamic opposition-based learning strategies, eliminating potential ambiguity regarding algorithmic innovations. We appreciate your guidance in strengthening manuscript clarity.

2.The section on Results in the abstract should include the necessary details. For example, authors described “The improved algorithm significantly outperformed other algorithms on 12 standard test functions.” Here, what are the improved algorithm and other algorithms? Authors should clearly specify the methods mentioned.

Dear Reviewer,

Thank you for your insightful suggestion to enhance methodological transparency. In the revised abstract, the Results section has been substantially expanded to explicitly specify all algorithms and models:

"The IBSLO demonstrated significantly superior convergence performance versus original BSLO, ant lion optimizer (ALO), Harris hawks optimization (HHO), and whale optimization algorithm (WOA) across all 12 CEC2022 test functions. Subsequently, the IBSLO-optimized automated machine learning (AutoML) model achieved ROC-AUC/PR-AUC values of 0.9038/0.8091 (training set) and 0.8775/0.8175 (testing set), significantly outperforming four baseline models: logistic regression (LR), support vector machine (SVM), XGBoost, and LightGBM. SHAP interpretability identified six key predictors: preoperative leukocyte count, body mass index (BMI), surgical approach, age, intraoperative blood loss, and C-reactive protein (CRP). Decision curve analysis demonstrated significantly higher net clinical benefit of the AutoML model compared to conventional methods across expanded threshold probability ranges (training set: 8–99%; testing set: 3–80%)."

This revision precisely delineates both the metaheuristic algorithms in functional benchmarking and the comparative prediction models, while maintaining conciseness through standardized abbreviation usage established in the Methods section.

3.Although authors mentioned the disadvantages of conventional machine learning algorithms, can the authors make comparisons between AutoML and those conventional machine learning algorithms (for example, random forest and support vector machines with similar tuning parameters)?

Dear Reviewer,

We thank the reviewer for this valuable comment and confirm that our study explicitly compared the AutoML framework with conventional machine learning methods. As documented in Table 2 of the manuscript, the AutoML model significantly outperformed support vector machines (SVM), logistic regression (LR), XGBoost, and LightGBM across multiple performance metrics. Specifically, on the training set, AutoML achieved superior ROC-AUC (0.9038 vs. SVM's 0.6752 and LR's 0.6843), PR-AUC (0.8091 vs. SVM's 0.4745), and F1-score (0.7771 vs. LightGBM's 0.6824). These quantitative comparisons demonstrate AutoML's enhanced capability in handling high-dimensional clinical data while addressing the limitations of manual hyperparameter optimization noted in conventional approaches.

4.Some technical terms may be unfamiliar to broad readers who lack a scientific understanding of machine learning methods. For example, what are the 12 CEC2022 test functions?

Dear Reviewer,

In response to the your insightful feedback regarding the accessibility of specialized machine learning terminology, we have expanded Section 2.3 to provide a comprehensive explanation of the CEC2022 test functions' purpose in algorithm benchmarking. This includes descriptions of their roles as standardized mathematical challenges for evaluating optimization performance across dimensions like uni/multi-modality, separability, and noise resistance. For complete transparency, we have additionally incorporated the full mathematical formulations of all 12 benchmark functions in Supplementary Material, enabling readers to verify the rigorous validation process applied to our enhanced IBSLO algorithm without requiring specialized computational expertise.

These enhancements collectively enhance methodological reproducibility while maintaining the technical integrity critical to computational science validation.

5.For independent t-tests, have the authors assessed the assumptions of the t-test?

Dear Reviewer,

We thank the reviewer for raising this important methodological consideration. We have now thoroughly addressed assumptions for independent t-tests in our revised statistical analysis section (Section 2.4). Specifically, all continuous variables underwent rigorous assessment for normal distribution prior to analysis. For variables satisfying normality assumptions and homogeneity of variance (verified via Levene's test), we proceeded with standard independent t-tests as indicated in the manuscript's cohort comparisons. These methodological refinements strengthen the validity of our cohort characteristic comparisons presented in Table 1, where all continuous features met parametric assumptions as confirmed by P-values exceeding 0.05 in both normality and homogeneity tests.

6.In the section “3.2 Algorithm Enhancement Performance”, how have the authors set up the 30 independent runs? The details need to be updated throughout the manuscript.

Dear Reviewer,

We appreciate the reviewer's valuable query regarding experimental reproducibility. Our revised Section 2.3 clarifies that all metaheuristic algorithm evaluations employed identical stochastic conditions: each algorithm completed 30 independent trials on each CEC2022 function with fixed-dimensional search spaces (D=10), constant population size (N=30), and uniform maximum iteration limits (T=500). To ensure statistical independence across runs, we implemented robust randomization protocols including unique seed initialization via MATLAB's rng('shuffle') command before each trial and dynamic opposition-based learning to diversify initial populations. The quantitative outcomes in Section 3.2 specifically report median fitness values and interquartile ranges derived from these 30 executions, with Figure 1's boxplots explicitly visualizing both solution precision (deviation from global optima) and algorithmic stability (variance across runs) for all benchmark categories.

7.The figures need to be revised with high-resolution ones. The figures are hard to read.

Dear Reviewer,

We sincerely thank the reviewer for bringing this crucial technical issue to our attention. We have comprehensively addressed the figure resolution requirements throughout the manuscript by regenerating all visual content using enhanced rendering techniques and standardized export protocols. These comprehensive enhancements collectively ensure optimal visual clarity during both digital viewing and print reproduction phases.

8.Authors should provide descriptions for each label in Figure 1. Additionally, the label on the x-axis should be presented consistently, using either a horizontal format or a 45-degree rotation.

Dear Reviewer,

We have implemented your valuable suggestions for improving Figure 1 as follows: The revised layout incorporates comprehensive data labels for all algorithm comparison groups (IBSLO, BSLO, ALO, HHO, WOA) using horizontal orientation throughout the x-axis for optimal readability. The updated version appears as Figure 1 in the manuscript (Section 3.2), where it continues demonstrating IBSLO's superiority in solution stability across benchmark problems while fully addressing the presentation issues raised. We confirm these modifications resolve the previous legibility challenges while preserving all original scientific insights and quantitative relationships.

9.I was unable to find the details of the results linked to the figures.

Dear Reviewer,

We appreciate the reviewer's diligent observation regarding figure-result correspondence and acknowledge that our initial description required enhanced clarity. In the revised Section 2.3, we have incorporated explicit interpretive guidance for algorithm testing visualizations.

This restructuring aligns with best practices in ML reporting while maintaining visual conciseness. We trust these additions will facilitate unambiguous interpretation of our model's analytical outputs and their clinical implications.

10. While the authors mentioned developing an intuitive risk prediction system in the manuscript, I could not find the link to the platform of the PPCs risk assessments (described in Figure 7)

Dear Reviewer,

We appreciate the your valuable query regarding the accessibility of our predictive system. The clinical decision support platform referenced in Figure 7 is currently deployed as a standalone MATLAB application rather than a web-based solution, developed using MATLAB App Designer (2024a) as explicitly noted in the Methodology section (Section 2.4) of our manuscript. This design choice prioritizes computational efficiency for real-time risk assessments and ensures robust data privacy compliance by processing inputs locally without transmitting sensitive patient data externally. While the desktop application requires installation of MATLAB Runtime, we acknowledge the convenience of browser-based access. Therefore, in response to this feedback: A cloud-based implementation leveraging TensorFlow.js is actively being developed for browser access.

Reviewer #3: Authors constructed an automated machine learning-based predictive model for postoperative pulmonary complication risk among non-small cell lung cancer patients who underwent thoracoscopic surgery. Leveraging automated machine learning in selecting significant features is interesting. However, there are several places where the manuscript can improve. I have suggested improvements to enhance the clarity of the reporting:

1. In the Abstract, authors reported “The AutoML model identified 5 important features: Preoperative leukocyte count; body mass index (BMI); Surgical approach; Age; Intraoperative blood loss; C-reactive protein (CRP).” The authors listed six features. Is it a typo?

Dear Reviewer,

We sincerely thank the reviewer for their meticulous attention to detail in identifying the numerical incongruity in our abstract statement. The sentence regarding feature quantity indeed contained a typographical error where we cited "5 important features" while enumerating six predictors: preoperative leukocyte count, BMI, surgical approach, age, intraoperative blood loss, and CRP. This inconsistency has been corrected throughout the manuscript to accurately reflect the SHAP analysis results, where six predictors were consistently identified as significant contributors to PPCs risk assessment.

2. How did you select six features? Have you used a fixed threshold based on the mean of SHAP or others for selecting features?

Dear Reviewer,

We appreciate the reviewer's insightful query regarding our feature selection methodology. The six predictive features were intrinsically selected through the AutoML framework's embedded feature engineering module, where our improved blood-sucking leech optimizer (IBSLO) algorithm simultaneously performed feature selection and hyperparameter tuning during model optimization without applying static SHAP-based thresholds. Specifically, this metaheuristic algorithm employed a multi-objective optimization approach to identify the feature subset maximizing predictive performance (quantified by cross-validated ROC-AUC) while maintaining clinical interpretability. The selection process occurred dynamically during model training through IBSLO's evolutionary operations, which evaluated feature subsets via 10-fold cross-validation on the training cohort. The final feature set emerged as the optimal combination that yielded maximal discriminative ability while balancing computational efficiency. Post-hoc SHAP analysis confirmed clinical relevance by ranking these exact features through their absolute mean SHAP value contributions but was not employed as a selection criterion. We have clarified this fully automated selection methodology in Section 2.3 of the revised manuscript, noting that "IBSLO's multi-criteria optimization simultaneously selected features while tuning hyperparameters based solely on cross-validated performance metrics."

3. Please describe the details of F1–F12 in Figure 1.

Dear Reviewer,

We acknowledge the reviewer's valuable request for additional technical details regarding the F1–F12 benchmark functions referenced in Figure 1. These functions constitute the standardized CEC2022 test suite employed for rigorous validation of our improved BSLO (IBSLO) algorithm. Specifically, the suite comprises 12 multidimensional optimization challenges categorized by complexity: F1–F3 represent unimodal landscapes (e.g., Shifted & Rotated Bent Cigar Function in F1) measuring exploitation efficiency; F4–F7 implement multimodal formulations testing global exploration and local minima avoidance; F8–F10 feature hybrid composite topologies combining multiple subfunctions with rotation matrices; while F11–F12 simulate real-world constrained engineering problems. In response to this query, we have explicitly elaborated in Section 2.3 (Study Design) that "all F1–F12 benchmarks were evaluated in 10-dimensional search spaces with predefined global optima to quantify IBSLO's convergence precision", including exemplar mathematical formulations. Furthermore, the complete function definitions with search space bounds and optima coordinates are now formalized in Supplementary, with derivations grounded in the CEC2022 technical report.

4. Did you use all variables in Table 1 for comparing the performance of IBSLO, BSLO, ALO, HHO, and WOA for Figures 1 and 2? Also, did you include all variables of Table 1 in the training and validation models?

Dear Reviewer,

We thank the reviewer for this important clarification regarding variable usage across different analytical phases. For Figures 1 and 2, which exclusively evaluate algorithmic performance, we benchmarked IBSLO against BSLO, ALO, HHO, and WOA using purely computational metrics derived from synthetic 10-dimensional CEC2022 test functions (F1–F12) without incorporating any clinical predictors. These tests measured metaheuristic convergence speed, solution precision, and optimization stability in isolation from clinical data. Conversely, for predictive model development, all demographic, metabolic-inflammatory, and surgical variables listed in Table 1 were incorporated during initial model training as potential predictors for the AutoML framework. The integrated feature selection mechanism within our IBSLO-enhanced AutoML autonomously identified the optimal feature subset during multi-objective optimization, ultimately reducing the initial variables to six clinically significant predictors through cross-validated performance maximization. This bifurcated approach—algorithm benchmarking versus predictive modeling—ensured rigorous technical validation of IBSLO’s mathematical properties before clinical application while maximizing discovery of biologically relevant predictors during model development. We have clarified these distinct analytical pipelines in Section 2.3: "Algorithm comparisons employed synthetic benchmark functions exclusively, whereas clinical predictor analysis utilized all available patient variables."

5. Please use the same scales on the y-axis on (A) and (B) of Figure 6 for comparison.

Dear Reviewer,

We sincerely appreciate the reviewer's valuable suggestion for scale standardization in Figure 6, which enhances its comparative utility. In response to this recommendation, we have meticulously standardized the y-axis scales identically across both subplots (A) and (B), maintaining a uniform net benefit range to facilitate direct visual cross-validation. Furthermore, as rigorously documented in our revised Results section, the updated analysis confirms that the AutoML model delivers significantly greater net therapeutic gains over an extended clinical applicability window: specifically, across risk thresholds spanning 8% to 99% for the training cohort and 3% to 80% for the testing cohort. These threshold bounds are now explicitly annotated in Figure 6 alongside the unmodified x-axis scope (0–100% probability), preserving methodological fidelity to DCA conventions while visually accentuating the model's broadened clinical utility beyond previous implementations.

6. How did you get 91.4% in Figure 7? Do you have any formulas for this calculation?

Dear Reviewer,

We sincerely appreciate the reviewer's discerning inquiry regarding the predictive probability calculation methodology. The 91.4% value displayed in Figure 7 is the real-time probabilistic output generated by our AutoML binary classification model, explicitly defined in the newly augmented "2.3 Study Design" section through the sigmoid transformation function represents the SHAP-derived model coefficients optimized via our IBSLO metaheuristic algorithm. This computational framework dynamically synthesizes six standardized preoperative predictors—including leukocyte count, BMI, and surgical approach—into a complication probability metric ranging between 0-100%. The specific 91.4% value emerges from the model's forward propagation when processing the case-specific input parameters visualized in Figure 7's interface. Crucially, this probability function constitutes the mathematical core of our clinical decision support system, transforming raw feature inputs into clinically actionable risk stratifications within the MATLAB App Designer environment. Its operational efficacy is formally validated through the model's maintained ROC-AUC of 0.8775 in independent testing, confirming reliability beyond the illustrated example.

7. Do you have any suggested percentages for close monitoring?

Dear Reviewer,

Thank you for your valuable input. We have implemented your recommendation by adding clinical monitoring thresholds in the Discussion section, specifying tiered protocols based on model-predicted risk probabilities: >70% (intensive monitoring), 40-70% (moderate monitoring), and <40% (standard monitoring) to enhance clinical applicability. This revision explicitly addresses resource allocation guidance while maintaining alignment with our DCA-inflected risk stratification strategy.

The added content concisely translates algorithmic outputs into actionable clinical pathways while reinforcing the study's translational value. We believe this strengthens the practical implementation framework of our CDSS.

8. Where are the results related to “the dynamic opposition-based learning strategy significantly improved local optimum avoidance rates”?

Dear Reviewer,

The empirical evidence substantiating the claim that "the dynamic opposition-based learning strategy significantly improved local optimum avoidance rates" is systematically presented in Section 3.2. Quantitative validation derives from 30 independent optimization runs on the CEC2022 benchmark suite, where convergence trajectory analysis (Figure 2) explicitly demonstrates IBSLO’s ability to evade local optima traps across all 12 test functions. This is particularly evident in complex multimodal landscapes like F7 and F11, where IBSLO maintained iterative solution diversity longer than the Harris Hawks Optimization comparator while reducing stagnation frequency. These performance metrics directly originate from the oppositional mechanism's mathematical formalism, which sustains directional exploration-exploitation balance through strategic position mirroring and stochastic perturbation.

9. Where are the results related to “particularly their superior capability in capturing nonlinear associations between features such as surgical approach and intraoperative blood loss compared to traditional logistic regression”?

Dear Reviewer,

Thank you for highlighting the need for explicit linkage between methodological claims and empirical results. We have revised the original statement to directly reference supporting evidence from our SHAP interpretability analysis and performance validation. The updated text now reads: "Our findings validate the restructured value of metaheuristic algorithms in medical data scenarios through their demonstrable superiority in capturing complex predictor interactions. These performance differentials originate from the algorithm's capacity to resolve high-dimensional interactions between surgical trauma and physiological reserves via iterative feature-space partitioning—a capability absent in generalized linear methodologies. "

10. SHAP interpretability analysis elucidated complex interaction patterns between key predictors and postoperative pulmonary complications (PPCs). Where are the results for the Analyzing feature interactions?

Dear Reviewer,

We thank the reviewer for prompting us to elaborate on the feature interaction analysis. As detailed in the newly added Figure 5(C) within section 3.5, the SHAP interaction heatmap explicitly quantifies nonlinear synergies between key predictors using pairwise SHAP interaction values. The heatmap's symmetrical matrix format further enables comparative assessment of interaction strengths through color intensity gradients, with the age-WBC and approach-CRP dyads exhibiting the strongest mutual reinforcement effects.

11. Did you find a U-shaped association in your data?

Dear Reviewer,

We are grateful for the reviewer's insightful critique regarding the interpretation of BMI's impact on postoperative outcomes, which prompted a rigorous re-evaluation of our analytical approach and narrative framing. In accordance with the methodological constraints identified by the reviewer—specifically the absence of demonstrable nonlinear associations in our dataset—we have comprehensively restructured the relevant discussion to eliminate any suggestion of U-shaped relationships or curvilinear continuity. This mechanistic reframing aligns precisely with our AutoML framework's identification of binary BMI thresholds as discrete risk modifiers rather than elements of a unified continuum, while avoiding speculative metabolic interpretations that exceeded the evidentiary boundaries of our feature interactions.
